# Functional mapping of the lower urinary tract by epidural electrical stimulation of the spinal cord in decerebrated cat model

**DOI:** 10.1038/s41598-024-54209-3

**Published:** 2024-04-26

**Authors:** Yuriy Sysoev, Elena Bazhenova, Polina Shkorbatova, Gleb Kovalev, Ivan Labetov, Natalia Merkulyeva, Dmitry Shkarupa, Pavel Musienko

**Affiliations:** 1 Pavlov Institute of Physiology, Russian Academy of Sciences (RAS), Saint-Petersburg, Russia; 2 Department of Neuroscience, Sirius University of Science and Technology, Sirius, Russia 354340; 3 Department of Pharmacology and Clinical Pharmacology, Saint-Petersburg State Chemical Pharmaceutical University, Saint-Petersburg, Russia; 4 Institute of Translational Biomedicine, Saint-Petersburg State University, Saint-Petersburg, Russia; 5 Saint-Petersburg State University Hospital, Saint-Petersburg, Russia; 6 Life Improvement by Future Technologies Center “LIFT”, Moscow, Russia 143025; 7 Center for Biomedical Engineering, National University of Science and Technology ”MISIS”, Moscow, Russia 119049

**Keywords:** Neural circuits, Somatosensory system, Spine regulation and structure

## Abstract

Several neurologic diseases including spinal cord injury, Parkinson’s disease or multiple sclerosis are accompanied by disturbances of the lower urinary tract functions. Clinical data indicates that chronic spinal cord stimulation can improve not only motor function but also ability to store urine and control micturition. Decoding the spinal mechanisms that regulate the functioning of detrusor (Detr) and external urethral sphincter (EUS) muscles is essential for effective neuromodulation therapy in patients with disturbances of micturition. In the present work we performed a mapping of Detr and EUS activity by applying epidural electrical stimulation (EES) at different levels of the spinal cord in decerebrated cat model. The study was performed in 5 adult male cats, evoked potentials were generated by EES aiming to recruit various spinal pathways responsible for LUT and hindlimbs control. Recruitment of Detr occurred mainly with stimulation of the lower thoracic and upper lumbar spinal cord (T13-L1 spinal segments). Responses in the EUS, in general, occurred with stimulation of all the studied sites of the spinal cord, however, a pronounced specificity was noted for the lower lumbar/upper sacral sections (L7-S1 spinal segments). These features were confirmed by comparing the normalized values of the slope angles used to approximate the recruitment curve data by the linear regression method. Thus, these findings are in accordance with our previous data obtained in rats and could be used for development of novel site-specific neuromodulation therapeutic approaches.

## Introduction

Storage and voiding are the main functions of lower urinary tract (LUT) that are under control of central nervous system pathways distributed throughout the brain and spinal cord^1^. LUT includes two main structures: bladder (detrusor muscle, Detr) and external urethral sphincter (EUS), which receives a bilateral innervation with autonomic and somatic fibers from the lumbosacral and thoracic segments of the spinal cord^2^. Preganglionic parasympathetic neurons are located in the conus medullaris, whereas preganglionic sympathetic neurons reside in the thoracolumbar spinal cord^3^. The spinal cord also has interneurons, which help to coordinate micturition reflex functions^4^. Detr relaxation and EUS tonic activity during the storage phase and Detr contractions and EUS relaxation during micturition are under control of pontine micturition center which coordinate spinal cord voiding reflexes^2^.

Several neurological diseases such as spinal cord injury (SCI), multiple sclerosis (MS) and Parkinson’s disease lead to LUT dysfunction^5,6^. The human EUS is normally relaxed during urination, otherwise there is a detrusor-sphincter dyssinergia (in the presence of confirmed neurological disease) or dysfunctional urination (in the absence of neurological disease)^7^. This condition creates increased pressure in the bladder, which over time can lead to vesicoureteral reflux. In addition, residual urine can exacerbate LUT infections^8,9^.

At present several neuromodulation techniques including sacral nerves^10,11^ or percutaneous tibial nerve^12,13^ stimulation have been developed in patients who failed conservative therapies, such as behavioral and pharmacological strategies. Although the current literature is optimistic about the use of the above-mentioned methods in the neurogenic population, the results are still inconsistent^14^. For example, existing studies of sacral neuromodulation are based on small sample sizes and heterogeneous populations that are not fully characterized in terms of severity of neurological impairment and lack standardized definitions of success and follow-up^15^. Worth to note that all proposed methods have several notable limitations and contraindications. For example, sacral neuromodulation may be associated with post-surgery complications, and is less effective in elderly population^16^. Another common problem of sacral neuromodulation is the loss of efficacy due to electrode migration^17,18^. However, there are approaches involving the use of transcutaneous electrical nerve stimulation (TENS) of the sacral nerves^19^, but their efficacy requires further research. One variation of TENS involves effects on peripheral nerves, such as tibial nerve stimulation. However, tibial nerve stimulation may not be suitable in patients with complete SCI because, as was evidenced by animal studies^20^, it requires intact supraspinal pathways.

A possible alternative or addition to the proposed methods could be a direct epidural electrical stimulation (EES)^21^. Unlike sacral neuromodulation or tibial nerve stimulation EES may directly influence the appropriate spinal cord segments. Clinical data and experimental results on animal models indicates that spinal cord stimulation can improve not only motor function but also ability to store urine and control micturition^22–24^. Decoding the spinal mechanisms that control the functioning of detrusor (Detr) and external urethral sphincter (EUS) muscles is essential for effective neuromodulation therapy in patients with disturbance of micturition. Presumably, EES restores neural control functions by delivering sub-motor threshold electrical impulses that transform the controlling neural networks into a more functional physiological state. To date EES was shown to be effective in treating both autonomic and motor disturbances in humans with SCI^25,26^. For effective application of EES, it is necessary to understand which regions of the spinal cord are responsible for activation of slow-contracting detrusor muscle and which for the fast-contracting EUS contractions. According to our previously obtained data, in rats detrusor muscle activation mainly occurs during the stimulation of the upper L1 and lower lumbar (L5–L6) spinal segments whereas EUS was activated predominantly by sacral stimulation^27^. Development of relevant experimental models for mapping the areas of the spinal cord responsible for urination is a priority task in the field of translational neurourology. Understanding the functions of LUT in animals will enable clinicians to treat patients with severe urinary dysfunction with more success. In continuation of our previous work^27^ in the present study we performed mapping of Detr and EUS by EES applying at different spinal cord regions (upper lumbar, lower lumbar and sacral) in decerebrated cat model. This unique model allowed inducing well-controlled reproducible locomotor behaviors and investigating the specific role of the spinal and brain stem networks in regulation of physiological functions including urinary system activity^28–30^. Decerebration, performed at different brain levels allows eliminating the influence of the rostrally located brain structures in order to explore the properties and capabilities of the spinal and brainstem structures located caudally to the site of transection. In addition, after decerebration there is no need to anesthetize the animal^31^, which is an undoubted advantage of this model for recording of evoked potentials since the possibility of the anesthetic influence is excluded. The obtained results give the green light to reveal the general patterns of LUT spinal control in different animal species.

## Results

### Evoked potentials in Detr, EUS and TA during 1 Hz stimulation

**Figure 1:**
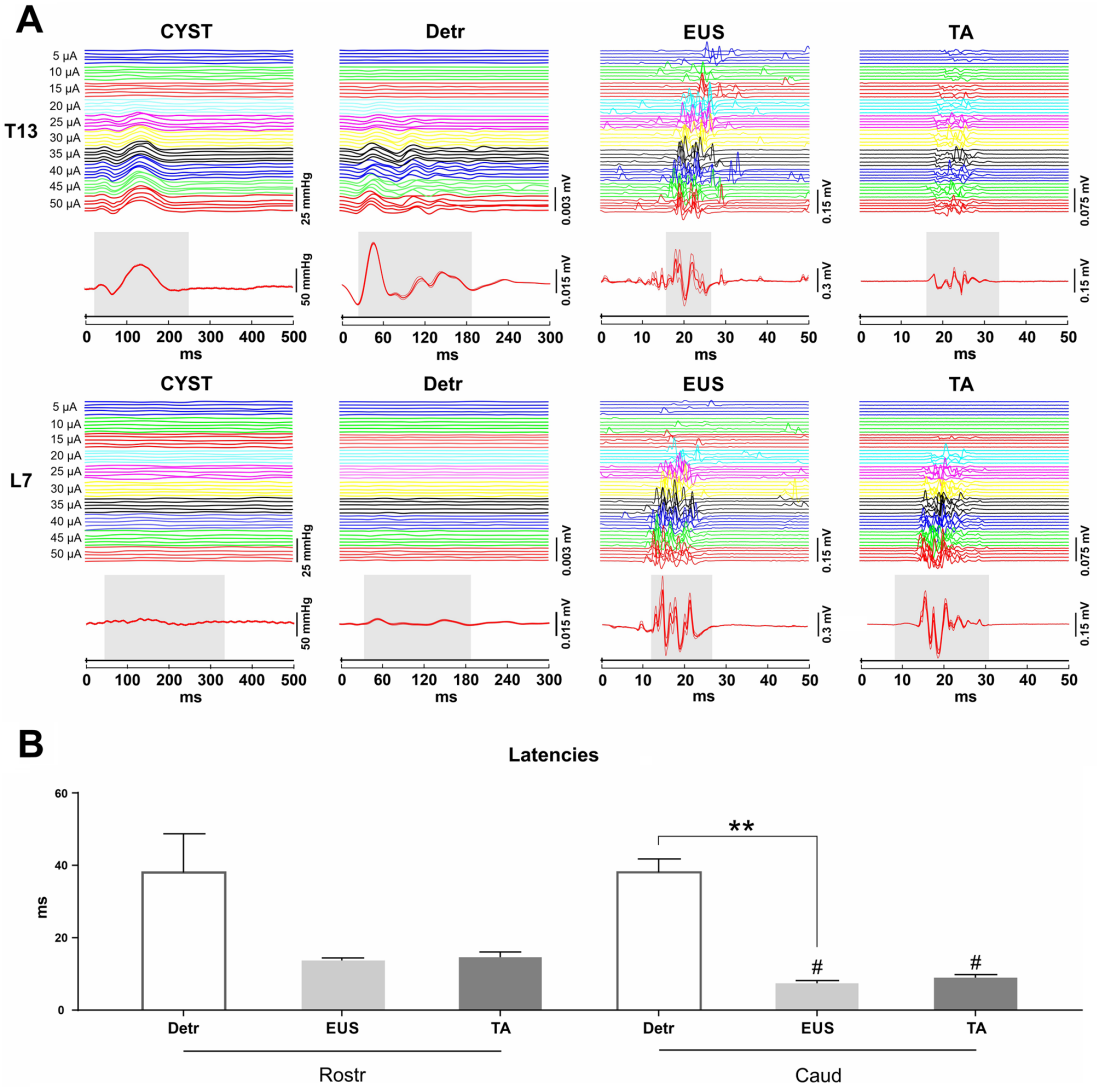
(**A**) Reflex recruitment in bladder pressure channel (CYST), detrusor (Detr), external urethral sphincter (EUS) and tibialis anterior muscle (TA) by the epidural spinal cord (T13 and L7 level) stimulation at a frequency of 1 Hz in decerebrated cat. Averaged evoked potentials (n = 5) in CYST, Detr, EUS and TA are presented for the maximum current (50 μA). Reflex responses are highlighted in gray. (**B**) Mean latencies of Detr, EUS and TA responses of rostral (Rostr, T13-L1) and caudal (Caud, L6-S1) levels of stimulation. Data are presented as mean ± SE, n = 3 for Rostr TA, n = 4 for Rostr Detr and EUS and Caud Detr, n = 5 for Caud EUS and TA. Note that in some cases, we could not define exact latency due to the background noise, or response was absent. **p < 0.01 – in comparison with Detr responses by Kruskal–Wallis test followed by Dunn's post-hoc test, ^#^p < 0.05 – in comparison with own values on the rostral level by Mann–Whitney test.

**Figure 2:**
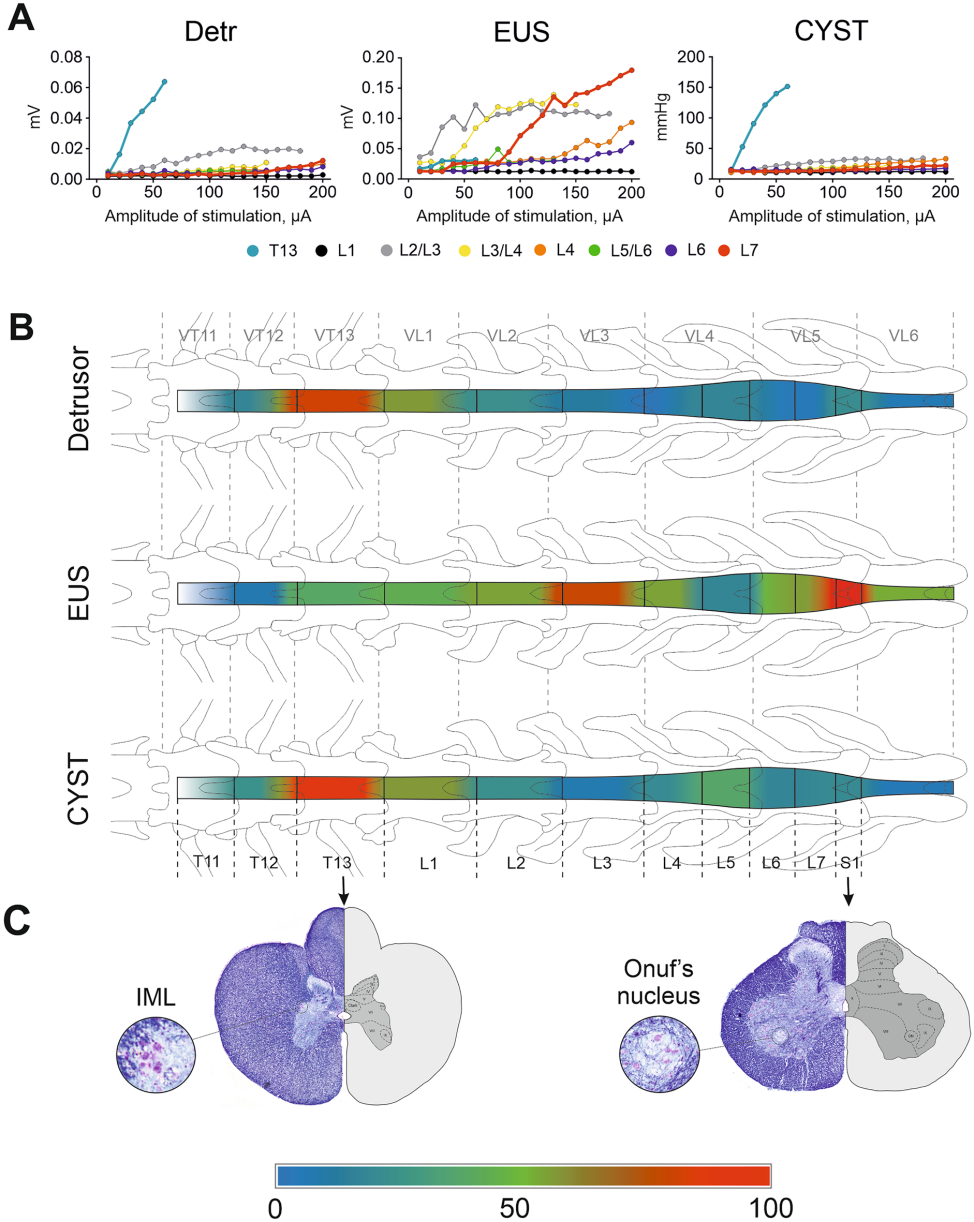
(**A**) Representative recruitment curves for detrusor (Detr), bladder pressure (CYST) and external urethral sphincter (EUS) during stimulation of different regions of the spinal cord in one cat. (**B**) Averaged normalized angles of slopes (in %) of Detr, EUS and CYST recruitment curves for various regions (segments) of the spinal cord in all cats (n = 5) presented as a heatmap, on the drawings of spinal cord. (**C**) Histological sections (Kluver-Barrera stain) and the scheme of gray matter structure of the spinal segments T13 with intermediolateral nucleus (IML) and S1 with Onuf’s nucleus.

Stimulation of lower thoracic (T13)/upper lumbar regions (L1) of the spinal cord triggered responses in all recorded channels (Figs. 1A, 2A,B). The Detr evoked potentials tended to have the longest latency (38.01 ± 9.58 ms) in comparison with EUS and TA muscles (13.72 ± 1.46 and 14.61 ± 2.50 ms, respectively, without statistical significance, Kruskal–Wallis test, H = 1.803, p = 0.4443) (Fig. 1B). The shape of Detr responses was represented by a slow wave of 200–250 ms duration that consisted of positive and negative peaks. CYST responses were also represented as slow waves with similar duration but unlike Detr muscle, they had fewer waves in their composition. EUS and TA responses were considerably faster and shorter and contained several positive and negative waves (Fig. 1A). In all channels, the observed responses were stable and their amplitude gradually increased with rising the magnitude of stimulation.

Application of EES at lower lumbar (L6-L7) or sacral (S1) level of the spinal cord evoked well-defined responses in EUS and TA muscles, whereas Detr and CYST waves had low amplitudes and were barely visible (Figs. 1A, 2A,B). In some animals reflex responses in EUS may have a lower threshold (vs lower thoracic/upper lumbar regions) but, eventually, the amplitude of the responses at the maximum current was higher. The latencies of EUS (7.41 ± 1.71 ms) reflexes were significantly shorter (H = 8.691, p = 0.0042 by Kruskal–Wallis test, p = 0.0117 by Dunn's post-hoc test) than the Detr responses (38.07 ± 7.37 ms) (Fig. 1B). Although TA responses (8.96 ± 1.99 ms) were also shorter in comparison with Detr reflexes there was no statistical significance there (p = 0.1065 by Dunn's post-hoc test). In addition, lower lumbar stimulation produced latencies in the EUS and TA that were shorter (p = 0.0159 and p = 0.0357, respectively, by Mann–Whitney test) than those observed during the upper lumbar stimulation. Noteworthy that latencies of Detr responses were almost similar (without significant difference, Mann–Whitney test) in both regions of the spinal cord. As was mentioned for lower thoracic/upper lumbar regions, in all channels the shape of the observed responses was stable, their amplitude increased as the stimulation magnitude rose until the submaximal level was achieved (Fig. 1A).

**Figure 3:**
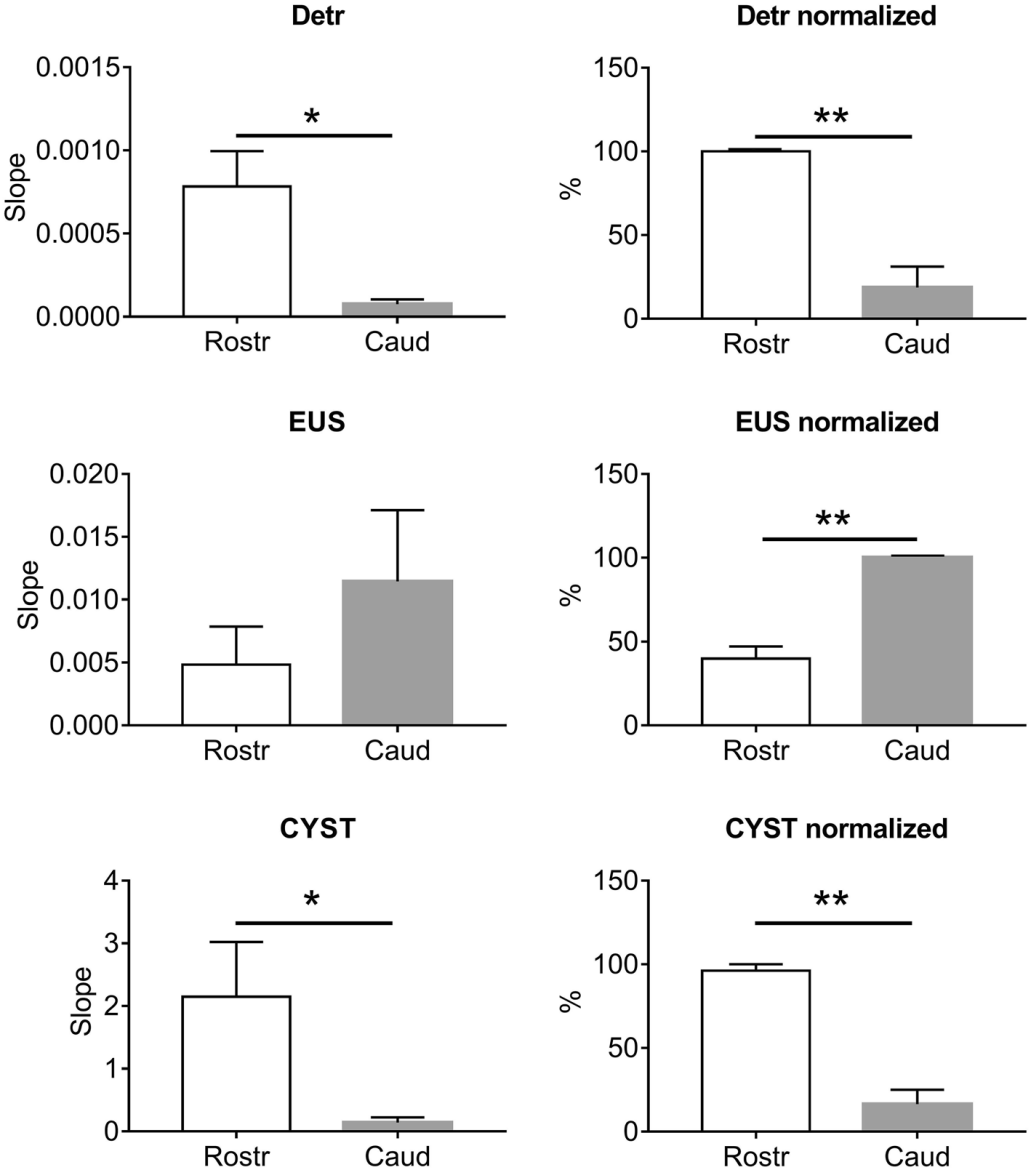
Averaged absolute and normalized values of recruitment curves slopes in rostral (Rostr, T13-L1) and caudal (Caud, L6-S1) segments. *Detr* detrusor, *CYST* bladder pressure, *EUS* external urethral sphincter. Data are presented as mean ± SE, n = 5 in all cases. *p < 0.05; **p < 0.01 by Wilcoxon test.

The comparison of the normalized values of the slope angles (Fig. 3) used to approximate the recruitment curve data by the linear regression method also indicated that recruitment of Detr and CYST occurred mainly with stimulation of the lower thoracic (T13) and upper lumbar spinal cord (L1). Responses in the EUS, in general, occurred with stimulation of various regions of the spinal cord, however, a pronounced specificity was evident for the lower lumbar (L6-L7) or upper sacral sections (S1).

### LUT activity during 5 Hz stimulation

**Figure 4:**
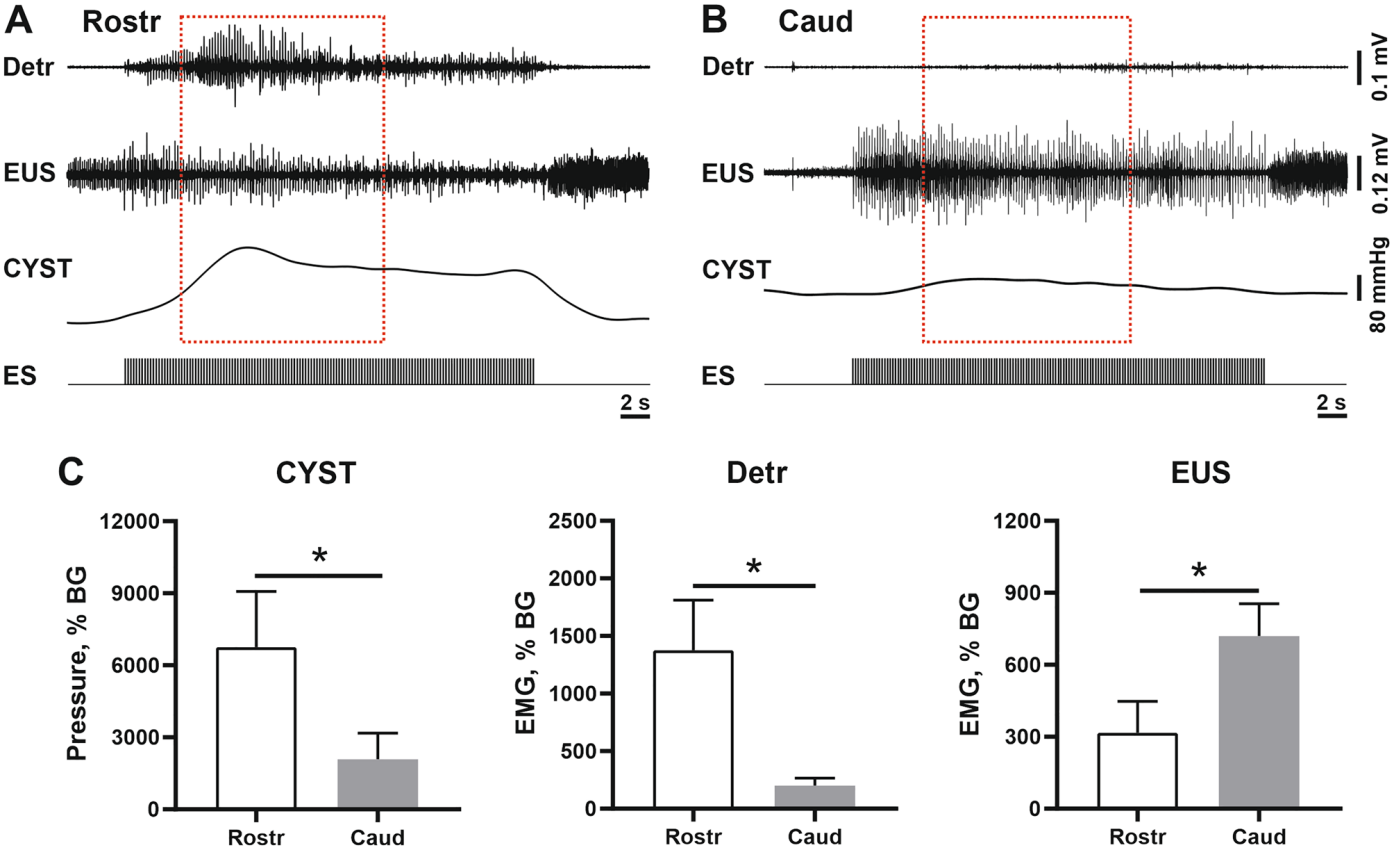
Representative examples of muscle responses in Detr, EUS and bladder pressure curve (CYST) during 5 Hz spinal cord epidural stimulation (ES). (**A**) ES of the rostral (Rostr) spinal region; (**B**) ES of the caudal (Caud) spinal region; (**C**) EMG amplitude of detrusor (Detr), external urethral sphincter (EUS), and bladder pressure (CYST) as a percentage to the signal before stimulation (“background”, BG). Data are presented as mean ± SE, n = 5 in all cases. Vertical scales at figures (**A**) and (**B**) are equalized. *p < 0.05 by Wilcoxon test.

Demonstrative examples of 5 Hz stimulation are shown at Fig. 4. The EES of the rostral (Rostr) region leads to the substantial increase of the EMG signal of the detrusor and the bladder pressure (Fig. 4A). In contrast, the EES of the caudal (Caud) region leads to the substantial increase of the EMG signal of the EUS (Fig. 4B). Percentage signals are presented at Fig. 4C. Both CYST and Detr signals had significantly higher (p = 0.0313 in both cases, Wilcoxon test) values during rostral stimulation – in all cats used (CYST: cat #101: 3077% vs 80%; cat #103: 4600% vs 3400%; cat #104: 9500% vs 1100%; cat #105: 14,500% vs 5700%; cat #106: 2070% vs 81%; Detr: cat #101: 1630% vs 170%; cat #103: 360% vs 180%; cat #104: 2050% vs 450%; cat #105: 2500% vs 120%; cat #106: 360% vs 85%). In contrast, EUS signal was higher during caudal stimulation – also in all cats used (cat #101: 555% vs 300%; cat #103: 850% vs 820%; cat #104: 270% vs 115%; cat #105: 900% vs 230%; #cat 106: 1010% vs 110%), these differences were also significant (p < 0.0313, Wilcoxon test). On average, the percentage values of the CYST signal during “optimal” stimulation site was higher compared with Detr and EUS signals; the lowest percentage signal was obtained for the EUS.

## Discussion

Improving the quality of life of patients with spinal cord injury is one of the priorities of medicine today^32–34^. In the United States, more than 288,000 people are living with spinal cord injuries, and approximately 17,700 new cases of SCI are reported each year^35^. In Saint Petersburg, Russia, the average annual incidence of TSCI was 17.6 per million, varying from 21.2 (2013) to 13.6 (2016)^36^. In addition to locomotor disorders, spinal cord injury patients suffer from sensory and autonomic disorders, including bowel problems, sexual and LUT dysfunctions^32^. It should be noted that mortality from life-threatening conditions such as acute kidney injury due to vesicoureteral reflux has been declining^37^. This is due to the expansion of treatment options, such as intermittent bladder catheterization, using indwelling catheters, condom catheter drainage, reflex voiding and bladder expression with Valsalva or Credé, oral drug therapy or Botulinum toxin A injections^38^. The most effective method for treatment of bladder dysfunction after SCI is intermittent self-catheterization in combination with anticholinergic medications^39^. 45 and 40% of male and female patients with SCI^35^ use this method. However, this approach is limited by the intolerance or lack of effectiveness of medications, high bladder pressure, persistent incontinence and lower urinary tract infections^39^. The side effects of anticholinergic drugs including constipation, dry month, blurred vision and headaches^40^ also reduce the quality of treatment. These therapy options do not provide the desired quality of life for patients with SCI, and restoring the full function of the LUT is still a problem. Due to these disadvantages, the importance of searching for novel therapeutic options in patients with LUT disturbances is undeniable.

### Spinal cord stimulation for treatment of LUT dysfunction

SCS has been shown to improve locomotor activity and function of the LUT after spinal cord injury^24,25^. It seems that locomotor activity in combination with SCS contributes to the improvement of LUT function. However, the mechanisms by which this effect is carried out are still not well understood. There are several approaches for placing electrodes for SCS near the spinal cord: subdural, epidural, transcutaneous or subcutaneous^21,41,42^. Some of these approaches, such as transcutaneous SCS and epidural SCS can be used in clinical practice^35^. In a report by Walter et al.^43^, lumbosacral epidural SCS has been shown to improve urinary and bowel function in a patient with spinal cord injury. Transcutaneous SCS is a non-invasive method which is comparable with epidural SCS. Several studies reported transcutaneous magnetic SCS improves bladder and bowel function^44,45^. However, the results were temporal and no subject maintained the capacity for voluntary micturition five weeks after the last effective stimulation. In study of Gad et al.^46^, transcutaneous SCS at T11 at 1 Hz improved voiding efficiency, increased flow rate, decreased residual volume and improved coordination between the detrusor and sphincter. Although spinal cord stimulation techniques for the treatment of LUT dysfunctions are not currently used as often in clinical practice compared to peripheral nerve stimulation techniques such as tibial and sacral neuromodulation, they may have potential in the future.

### Neuronal pathways underlying the reflex responses in Detr and EUS during the EES

The present study demonstrates that stimulation on the lower thoracic/upper lumbar level of the spinal cord triggers responses predominantly in Detr and CYST channels whereas lower lumbar/sacral regions predominantly activate reflexes in EUS. These observations are similar to our previously published rat data where activation of detrusor muscle mainly occurred during the stimulation of the upper lumbar (L1) and lower lumbar (L5-L6) spinal segments whereas the external urethral sphincter was activated predominantly by sacral stimulation^27^.

Unlike other visceral systems (e.g. gastrointestinal or cardiovascular) LUT function is highly dependent on the central nervous system pathways. The neural control of micturition is organized as a hierarchical system in which spinal urine storage mechanisms are regulated by descending projections of rostral brainstem circuits. It is noteworthy that effective micturition requires the integration of autonomic and somatic efferent mechanisms to coordinate the reciprocal activity of Detr and EUS^1,47^. In cats sympathetic innervation of LUT originates from intermediolateral nuclei (Fig. 2C) in the thoracic and upper lumbar segments of the spinal cord^48^ and runs through the inferior mesenteric plexus and the hypogastric nerves to the base of the bladder and the urethra. Sympathetic postganglionic neurons release norepinephrine, which activates β3 adrenergic receptors to relax Detr muscle and activates α1 adrenergic receptors to contract the internal urethral sphincter. Parasympathetic preganglionic fibers arise from cell bodies located in a ventrolateral band within the sacral parasympathetic nuclei of the intermediate gray matter of the S1-S3 spinal segments and travel in sacral roots and pelvic nerves to ganglia in the pelvic plexus and in the bladder wall. Parasympathetic postganglionic axons of the pelvic nerve release acetylcholine, which causes a Detr contraction by stimulating M3 muscarinic receptors in the bladder smooth muscle. The somatic efferent fibers innervate EUS and pelvic floor via the pudendal nerve, mediating striated muscle contraction by activation of nicotinic receptors. These cholinergic motor neurons (forming well-known Onuf’s nuclei, Fig. 2C) are located in the ventral horns on S1-S2 spinal level^48^. In general, the observed results correspond to nuclei distribution in cat spinal cord, however there were some differences of “hot points” localization between individual cats. These variations could be attributed to the different mechanisms of spinal cord activation by EES. For example, the first of them is associated with the activation of the afferent pathways of the dorsal columns and the antidromic distribution of current along their fibers to ramifications, which have monosynaptic switching on motoneurons. The second one may occur due to the excitation of the afferents of the dorsal roots located next to the stimulated electrode, and the orthodromic distribution of the current to monosynaptic switching on motor neurons. The last mechanism of activation explains why stimulation of caudal segments of the sacral region (or even coccygeal level) could activate EUS responses. It is also necessary to take into account the individual variability in the location of the nuclei and segments of the feline spinal cord relative to the vertebrae, which was shown in previously published articles^1,47–49^.

### Site-specific modulation of LUT by EES

Acute EES of the L3 segment in rats was previously reported to relax the urethra and facilitate urination^50,51^. It was shown that during stimulation tonic activity was suppressed, and then the bursting responses were evoked^50^.

The rat exhibits tonic EUS contractions during the bladder filling phase, and the EUS switches to a bursting pattern, which consists of intermittent periods of relaxation and phasic activation, during voiding^51^. The motoneurons of the pudendal nerve, the fibers of which innervate the EUS, are located in the dorsolateral nucleus of L6-S1. There are studies suggesting that tonic and bursting activity may be mediated by circuitry in the lumbosacral region of the spinal cord^52,53^. Other studies in rats have shown that the bursting activity of EUS during urination in rats is generated at the L3-L4 segmental levels of the spinal cord^1,54^.

According to acute studies in cats, Detr contraction can be induced by electrical stimulation of the ventral roots of the sacral parasympathetic segmental levels^55,56^. Moreover, microstimulation with a stimulus intensity of 100 µA and a duration of 30–60 s using a single microelectrode in the S2 lateral ventral horn or ventral funiculus induced high-amplitude bladder contractions with small urethral contractions^56^. In addition, it has been shown that partial relaxation of the EUS can be caused by microstimulation of the dorsal gray commissure^57^. In another study, Pikov et al.^58^ used intraspinal (dorsal horn) stimulation of the sacral spinal cord for bladder voiding in cats before and after T12 transection SCI. Preganglionar neurons and pathways as well as motorneurons in Onuf’s nuclei are located in a close relation to the caudal stimulation sites and the neuromodulation effect is depended by the degree of concurrent and synergistic activations of Detr vs EUS associated functional networks. Thus, there is significant debate, what fibers are activated by SCS and how different fibers activation varies for the different waveform patterns and intensity^59^. It is also unclear which patterns should be activated for achieving specific effects: chronic pain relief^60,61^, activating Detr and EUS contractions or inhibiting it.

Epidural stimulation of T13 spinal cord level triggered responses in both detrusor and EUS muscles resulting in extremely high bladder pressure responses (> 50 mmHg or > 68 cmH_2_0) (Fig. 1A). This is in accordance with recent SCS studies in SCI human subjects where voiding was induced with a high bladder pressure (> 50 cmH2O or almost 100 cmH2O) indicating a co-activation of detrusor and EUS^62^. High pressure voiding mediated by bladder contraction against the closed urethra is harmful to the kidney function due to possible vesicoureteral reflux^63^. Subsequent renal failure after long-term clinical application is a critical problem preventing the SCS from clinical application at this time. However, recent chronic SCI cat study indicates that 10 kHz bilateral pudendal nerve stimulation (PNS) can relax the EUS and reduce the urethral outlet resistance^64^. Taking into account our results, it is possible to suggest that combination of SCS and PNS can lead to restoration of bladder function after SCI without renal impairment. Moreover, unlike PNS alone, combination with SCS could allow to achieve not only successive voiding, but also improvement of locomotor, postural, sexual and bowel function^25,65^. There is increasing evidence to suggest that electrical modulation promotes neuroregeneration and neural repair by affecting signaling in the nervous system. SCS with motor skill rehabilitation training makes use of residual nerve fibers for collateral growth, encourages the formation of new synaptic connections to promote neural plasticity, and improves motor function recovery in patients with spinal cord injury^66^.

As the exact mechanisms of SCS action still needs to be investigated^35^, the identification of optimal or even appropriate stimulation parameters and their translation between animal models and humans remains challenging. It should be determined if the same stimulation parameters can be used for the treatment of multiple dysfunctions (i.e. locomotor and postural recovery versus restoration of bladder function) or if different stimulation parameters will be required. Worth to mention that the most effective stimulation parameter may vary from subject-to-subject, and stimulation parameters that have been configured for one bladder function (e.g. storage) may not be suitable for another (e.g. voiding)^58,65^. To date in clinical practice stimulation frequencies range from 5 to 40 Hz^25,65^ but final success of rehabilitation also depends on appropriate duration of stimulation session and presence of locomotor training. Recruitment at 1 Hz mapping may suggest possible pathways that are involved during the stimulation of a particular area of the spinal cord; however, the functional significance of stimulation of selected areas of the spinal cord requires future experimental confirmation.

## Conclusions

The obtained results demonstrate that in decerebrated cat recruitment of Detr occurs mainly with stimulation of the lower thoracic and upper lumbar spinal cord (T13-L1). In contrast, EUS activation could be initiated with stimulation of all the studied sites of the spinal cord, however, a pronounced specificity is noted for the lower lumbar/upper sacral sections (L7-S1). These findings are in accordance with our previous data obtained in rats which may indicate that this site-specificity is typical, in general, in all mammals. Understanding of localization of spinal networks, responsible for selective activation of Detr or EUS is an important component for development of novel site-specific neuromodulation therapeutic approaches.

## Methods

### Animals

The study was performed in 5 adult male cats (#101, #103, #104, #105 and #106) weighing 3–5 kg. The animals were bred and housed at the animal facility of the Pavlov Institute of Physiology. All cats were housed separately at a room temperature of 23 ± 1 °C with ad libitum access to food and water. The Ethics Commission of the Pavlov Institute of Physiology approved all experimental procedures (protocol #01/2020). Experiments were performed in accordance with requirements of Council Directive 2010/63EU of the European Parliament on protection of animals used in experimental and other scientific purposes. The number of animals used, and their pain and distress were minimized. Referring to our previously published report that have recorded EES evoked potentials in a similar number of animals^27^, we deemed our sample size sufficient to evaluate our model. The study is reported in accordance with the ARRIVE guidelines (https://arriveguidelines.org).

### Surgery

**Figure 5:**
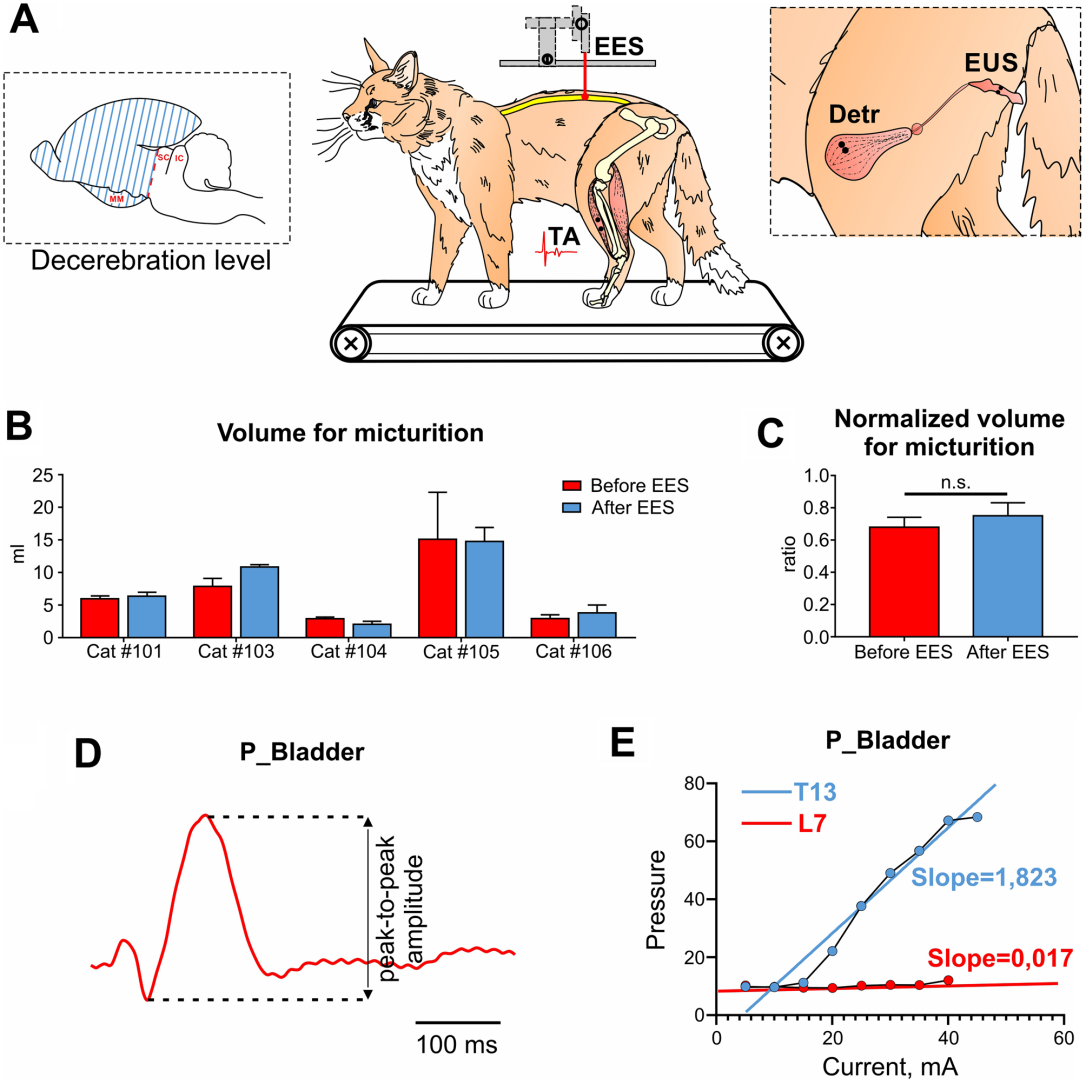
(**A**) Decerebrated cat model to investigate the effect of spinal cord epidural electrical stimulation (EES) to the lower urinary tract (LUT) system. EMG electrodes were implanted in the external urethral sphincters (EUS), Detrusor (Detr) and tibialis anterior (TA) muscles. *MM* mammillary bodies, *SC* superior colliculus, *IC* inferior colliculus. (**B**) Normalized values of volumes of the infused saline to initiate micturition before and after EES mapping. In each cat 2–3 volumes before and after EES mapping were normalized to the maximum value; (**C**) Mean values of volume of the infused saline to initiate micturition in all tested cats (n = 5). *n.s*. non-significant difference by paired Wilcoxon test; (**D**) Example of CYST response (pressure evoked potentials during 1 Hz stimulation); (**E**) Calculation of the linear regression function slope of obtained recruitment curves. The calculation of the linear regression function was carried out according to the recruitment curves (built on the red or blue dots) until the maximum values were reached. The red and blue lines represent the plots of the linear regression functions.

After preliminary injection of xylazine (2 mg/kg) the animals were anesthetized by inhalation of isoflurane (1.5–2.5%) with oxygen. The level of anesthesia was controlled by tests for paw sensitivity to mechanical pressure of the skin as well as by checking the reaction of the pupils to the light. The head and spinal column were rigidly fixed in the metal frame with paws standing on the treadmill belt (flat surface). Then after the ligation of the common carotid arteries and the craniotomy, we performed precollicular–postmammillary decerebration (Fig. 5A). The level of decerebration was verified after the experiment with dissection of the brainstem. After decerebration, a median dorsal skin incision was made on the back and interlaminectomies were performed between each of the lower thoracic (VT11-VT13) and lumbar (VL1-VL6) vertebrae. The effect of anesthesia ceased after surgical interventions, and the experiments began 1–2 h after decerebration. The rectal temperature, arterial pressure, electrocardiographic and breathing rates were continuously monitored during the experiment. The intravesical pressure was measured using a cystometry (CYST) sensor. Following a midline abdominal incision, two catheters (Perifix 401, 18G) were introduced through the apex of the bladder and secured using a 6.0 Ethilon suture (Ethicon, New Brunswick, NJ). One of them was connected to a solid-state pressure transducer (MLT0670, AU) to record the intravesical pressure and the other was used to fill the bladder with the room temperature 0.9% saline solution through syringe pump (ZOOMED SN-1600 V, RU). Bipolar electromyographic electrodes (0.2 mm flexible stainless-steel Teflon-insulated wires) were implanted into detrusor, EUS and m. tibialis anterior (TA, ankle flexor). The urethra was not closed and the urination occurred in a natural manner. The outer part of the urethra was placed in a funnel-like urine collector from which urine flowed into a measuring cup. At the termination of the experiments, the cats were euthanized with overdose of isoflurane (5%), and then perfused transcardially with isotonic saline followed by 4% paraformaldehyde solution. Then a detailed dissection of vertebrae, roots, and spinal cord was performed to determine the exact level of the spinal cord stimulation, including laminectomies and the spinal segments^67^.

### Epidural electrical stimulation

Evoked potentials were generated by EES (Fig. 5A) with a monopolar silver ball electrode (d = 0.5 mm, 1 Hz frequency at stimulation intensities ranging from 10 µA to 800 µA in increments of 10 µA, 10 pulses for each stimulation amplitude, pulse duration of 0.3 ms) aiming to recruit various spinal pathways responsible for LUT and hindlimbs control in upper lumbar, lower lumbar, sacral, and coccygeal spinal cord regions. Two regions of interest were used: (1) rostral region corresponding to the caudal thoracic (T12-T13) and rostral lumbar (L1-L2) segments; (2) caudal region corresponding to the caudal lumbar (L6), sacral segments, and coccygeal segment Co1. We also assessed a 5 Hz electrical stimulation evoked EMG signals as a percentage to the signals before stimulation (“background”, BG activity). The precise identification of the stimulating points was carried out post-mortem on the base of the interroot-root variant of spinal cord segmental division^68^. The reference electrode made of a 21G needle was placed in paravertebral muscle similar to other studies in this model^29^. To be sure that the bladder state was constant during the experiment, before and after testing of the reflex responses to EES the urodynamic studies were performed. The bladder catheter was connected to the infusion pump to infuse the bladder with sterile saline at a rate of 3 ml/min. In each cat, we analyzed the storage volume (volume of infused saline to initiate micturition). For this, we performed 2–3 cycles of infusion/micturition. (Fig. 5B,C).

The EMG electrodes signals were differentially amplified (A-M Systems, model 1700, US, bandwidth of 10 Hz to 5 kHz), digitized at 20 kHz with a National Instrument A/D board, rectified, and integrated by computer programs. Custom scripts written in Matlab were used to measure evoked potentials from the selected muscles. We analyzed latency (the first peak) in Detr, EUS, TA (Fig. 1) and peak-to-peak amplitude of the first peak of responses (Fig. 5D) in Detr, EUS and CYST to build the recruitment curves (Fig. 5E) for each stimulation point (Fig. 2A). For each recruitment curve, the corresponding slope of the linear regression function (Fig. 5E) was calculated. The values obtained for each stimulation point were normalized relative to the maximum and then averaged.

### Statistical analysis

The data are presented as mean ± standard error (SE). Statistical significance was assessed using the Mann–Whitney test – in the case of non-paired comparisons (see Fig. 1B, rostral vs caudal level), or using paired Wilcoxon test – in the case of paired comparisons (see Figs. 3, 4C). The mean latencies of Detr, EUS and TA responses were compared with Kruskal–Wallis test followed by Dunn's post-hoc test (see Fig. 1B). Intergroup differences were considered statistically significant at p < 0.05. 

## Data Availability

The datasets analyzed during the current study are available from the corresponding author on reasonable request.
